# Correction: The use of bluetooth low energy Beacon systems to estimate indirect personal exposure to household air pollution

**DOI:** 10.1038/s41370-020-0215-5

**Published:** 2020-02-27

**Authors:** Jiawen Liao, John P. McCracken, Ricardo Piedrahita, Lisa Thompson, Erick Mollinedo, Eduardo Canuz, Oscar De Léon, Anaité Díaz-Artiga, Michael Johnson, Maggie Clark, Ajay Pillarisetti, Katherine Kearns, Luke Naeher, Kyle Steenland, William Checkley, Jennifer Peel, Thomas F. Clasen, HAPIN investigators, Vigneswari Aravindalochanan, Kalpana Balakrishnan, Dana Boyd Barr, Vanessa Burrowes, Devan Campbell, Julia McPeek Campbell, Adly Castañaza, Howard Chang, Yunyun Chen, Marilú Chiang, Rachel Craik, Mary Crocker, Victor Davila-Roman, Lisa de las Fuentes, Ephrem Dusabimana, Lisa Elon, Juan Gabriel Espinoza, Irma Sayury Pineda Fuentes, Sarada Garg, Dina Goodman, Savannah Gupton, Stella Hartinger, Steven Harvey, Mayari Hengstermann, Phabiola Herrera, Shakir Hossen, Penelope Howards, Lindsay Jaacks, Shirin Jabbarzadeh, Abigail Jones, Miles Kirby, Jacob Kremer, Margaret Laws, Amy Lovvorn, Fiona Majorin, Eric D. McCollum, Rachel Meyers, J. Jaime Miranda, Lawrence Moulton, Krishnendu Mukhopadhyay, Abidan Nambajimana, Florien Ndagijimana, Azhar Nizam, Jean de Dieu Ntivuguruzwa, Aris Papageorghiou, Naveen Puttaswamy, Elisa Puzzolo, Ashlinn Quinn, Sarah Rajkumar, Usha Ramakrishnan, Davis Reardon, Ghislaine Rosa, Joshua Rosenthal, P. Barry Ryan, Zoe Sakas, Sankar Sambandam, Jeremy Sarnat, Suzanne Simkovich, Sheela Sinharoy, Kirk R. Smith, Damien Swearing, Gurusamy Thangavel, Ashley Toenjes, Lindsay Underhill, Jean Damascene Uwizeyimana, Viviane Valdes, Amit Verma, Lance Waller, Megan Warnock, Kendra Williams, Wenlu Ye, Bonnie Young

**Affiliations:** 1 Department of Environmental Health, Emory University, Atlanta, GA USA; 2 Centro de Estudios en Salud, Universidad del Valle de Guatemala, Guatemala City, Guatemala; 3 Berkeley Air Monitoring Group, Berkeley, CA USA; 4 Nell Hodgson Woodruff School of Nursing, Emory University, Atlanta, GA USA; 5 Department of Environmental and Radiological Health Sciences, Colorado State University, Fort Collins, CO USA; 6 Environmental Health Sciences, School of Public Health, University of California, Berkeley, CA USA; 7 College of Public Health, University of Georgia, Athens, GA USA; 8 Division of Pulmonary and Critical Care, School of Medicine, Johns Hopkins University, Baltimore, MD USA; 9 Center for Global Non-Communicable Diseases, Johns Hopkins University, Baltimore, MD USA; 10 Sri Ramachandra Institute of Higher Education and Research, Chennai, India; 11 School of Medicine, Johns Hopkins University, Baltimore, MD USA; 12 Rollins School of Public Health, Emory University, Atlanta, GA USA; 13 A.B. PRISMA, San Miguel, Peru; 14 University of Oxford, Oxford, UK; 15 Seattle Children’s Hospital, Seattle, USA; 16 Washington University in St. Louis, St. Louis, MO USA; 17 Eagle Research Center, Kigali, Rwanda; 18 Universidad Peruana Cayetano Heredia, Lima, Peru; 19 Johns Hopkins Bloomburg School of Public Health, Johns Hopkins University, Baltimore, MD USA; 20 Freie Universitat Berlin, Berlin, Germany; 21 Johns Hopkins University, Baltimore, MD USA; 22 T.H. Chan School of Public Health, Harvard University, Boston, MA USA; 23 London School of Hygiene and Tropical Medicine, London, UK; 24 School of Medicine, Universidad Peruana Cayetano Heredia, Lima, Peru; 25 Johns Hopkins Bloomberg School of Public Health, Johns Hopkins University, Baltimore, MD USA; 26 Global LPG Partnership, New York, NY USA; 27 Fogarty International Center, National Institutes of Health, Bethesda, MD USA

**Correction to: Journal of Exposure Science & Environmental Epidemiology**

10.1038/s41370-019-0172-z

In the original Article, Eric D. McCollum’s name was incompletely stated (as Eric McCollum). This has been corrected in the HTML, PDF and XML versions of this article.

